# Supplementary material for: Effect of Substitution Degree and Homogeneity on Cyclodextrin-Ligand Complex Stability: Comparison of Fenbufen and Fenoprofen Using CD and NMR Spectroscopy
Source: Int J Mol Sci. 2023 Apr 19;24(8):7544. doi: 10.3390/ijms24087544 (PMC10139022; doi:10.3390/ijms24087544)
Supplement: Supplementary file 1 [file ijms-24-07544-s001.zip › ijms-2348295-supplementary.pdf]

Supplementary Material

Effect of substitution degree and homogeneity on cyclodextrin-ligand complex stability: comparison of fenbufen and fenoprofen using CD and NMR

Márta Kraszni[1], Ferenc Ágh[1], Dániel Horváth[2], Arash Mirzahosseini[1], Péter Horváth[1]*

[1]Department of Pharmaceutical Chemistry, Semmelweis University, Hőgyes Endre utca 9, 1092 Budapest, Hungary

[2]ELKH-ELTE Protein Modelling Research Group, Eötvös Loránd University, Pázmány Péter sétány 1A, 1117 Budapest, Hungary

*to whom correspondence should be addressed:
Dr. Péter Horváth
Department of Pharmaceutical Chemistry, Semmelweis University
H-1092 Budapest, Hőgyes E. u. 9, Hungary
Phone/Fax: +3612170891
E-mail: horvath.peter@semmelweis.hu

Table S1. The measured ellipticity of fenbufen by the addition of different amount of beta-cyclodextrin (BCyD), randomly methylated methyl-beta-cyclodextrin (CRYSMEB) and random methyl-beta-cyclodextrin (RAMEB).

$c_{fenbufen}$ (mM)	c_{BCyD} (mM)	$\theta_{284.2\ nm}$ (mdeg)	$c_{fenbufen}$ (mM)	$c_{CRYSMEB}$ (mM)	$\theta_{280.8\ nm}$ (mdeg)	$c_{fenbufen}$ (mM)	c_{RAMEB} (mM)	$\theta_{282.6\ nm}$ (mdeg)
0.985	0	0	0.985	0	0	1.038	0	0
	0.293	2.18		0.255	1.96		0.220	1.57
	0.586	4.07		0.509	2.93		0.513	3.39
	1.76	8.44		1.53	7.12		1.32	6.86
	2.93	9.59		2.55	8.67		2.49	8.83
	5.86	10.89		5.10	10.15		4.98	9.76
	11.7	11.32		10.2	10.26		10.0	10.09
	17.6	11.61		15.3	9.94		15.1	10.30
0.971	23.1	11.59	0.972	20.1	10.12		19.8	10.36

Table S2. The measured ellipticity of fenbufen by the addition of different amount of methyl-beta-cyclodextrins (DIMEB50, DIMEB80, DIMEB95).

$c_{fenbufen}$ (mM)	$c_{DIMEB50}$ (mM)	$\theta_{282.7\ nm}$ (mdeg)	$c_{fenbufen}$ (mM)	$c_{DIMEB80}$ (mM)	$\theta_{282.8\ nm}$ (mdeg)	$c_{fenbufen}$ (mM)	$c_{DIMEB95}$ (mM)	$\theta_{282.8\ nm}$ (mdeg)
0.985	0	0	0.985	0	0	0.985	0	0
	0.244	3.09		0.254	4.02		0.254	4.02
	0.488	4.32		0.508	5.09		0.508	5.09
	1.47	9.03		1.53	10.32		1.53	10.32
	2.44	10.69		2.54	11.56		2.54	11.56
	4.88	11.36		5.08	12.44		5.08	12.44
	9.77	11.16		10.2	12.01		10.2	12.01
	14.7	11.48		15.2	11.38		15.2	11.38
0.972	19.3	11.04	0.972	20.1	11.40	0.972	20.1	11.40

Table S3. The measured ellipticity of fenbufen by the addition of different amount of hydroxypropyl-beta-cyclodextrins (HPBCyD(4.5), HPBCyD(6.3)).

$c_{fenbufen}$ (mM)	$c_{HPBCyD(4.5)}$ (mM)	$\theta_{282.7\ nm}$ (mdeg)	$c_{fenbufén}$ (mM)	$c_{HPBCyD(6.3)}$ (mM)	$\theta_{283.0\ nm}$ (mdeg)
1.038	0	0	1.038	0	0
	0.226	1.62		0.218	1.62
	0.564	3.04		0.544	3.11
	1.02	5.36		0.979	4.92
	1.58	6.61		1.52	6.74
	2.59	7.75		2.50	7.73
	5.19	8.81		5.00	8.33
	10.3	9.19		9.90	8.74
	15.5	9.34		14.9	8.81
	20.3	9.72		19.6	8.94

Table S4. Chemical shift change of fenbufen aromatic hydrogens induced by complexation with beta-cyclodextrin (BCyD).

$c_{fenbufen}$ (mM)	c_{BCyD} (mM)	$\Delta\delta_{free} - \Delta\delta_{complexed}$ (ppm)			
		H2,2'	H3,3'	H4,4'	H5
0.985	0.293	-0.032	-0.040	-0.002	0.006
	0.586	-0.061	-0.080	-0.014	-0.002
	1.76	-0.132	-0.192	-0.072	-0.040
	2.93	-0.154	-0.230	-0.095	-0.062
	5.86	-0.167	-0.251	-0.108	-0.071
	11.7	-0.172	-0.259	-0.116	-0.078
	17.6	-0.174	-0.263	-0.118	-0.082
0.971	23.1	-0.174	-0.263	-0.118	-0.089

Table S5. Chemical shift change of fenbufen aromatic hydrogens induced by complexation with randomly methylated methyl-beta-cyclodextrin (CRYSMEB).

$c_{fenbufen}$ (mM)	$c_{CRYSMEB}$ (mM)	$\Delta\delta_{free} - \Delta\delta_{complexed}$			
		H2,2’	H3,3’	H4,4’	H5
0.985	0.255	-0.031	-0.033	0.004	0.011
	0.509	-0.038	-0.040	0.004	0.012
	1.53	-0.124	-0.128	0.018	0.045
	2.55	-0.151	-0.155	0.023	0.056
	5.10	-0.169	-0.172	0.027	0.064
	10.2	-0.176	-0.176	0.027	0.066
	15.3	-0.177	-0.177	0.028	0.067
0.972	20.1	-0.177	-0.177	0.027	0.067

Table S6. Chemical shift change of fenbufen aromatic hydrogens induced by complexation with methyl-beta-cyclodextrin (DIMEB50).

$c_{fenbufen}$ (mM)	$c_{DIMEB50}$ (mM)	$\Delta\delta_{free} - \Delta\delta_{complexed}$			
		H2,2’	H3,3’	H4,4’	H5
0.985	0.244	0.004	0.004	-0.053	0.010
	0.488	0.004	-0.078	-0.076	0.013
	1.47	-0.002	-0.160	-0.155	0.031
	2.44	-0.005	-0.186	-0.179	0.036
	4.88	-0.007	-0.199	-0.188	0.031
	9.8	-0.008	-0.199	-0.186	0.033
	14.7	-0.009	-0.197	-0.186	0.033
0.972	19.3	-0.009	-0.195	-0.184	0.034

Table S7. Chemical shift change of fenbufen aromatic hydrogens induced by complexation with methyl-beta-cyclodextrin (DIMEB80).

$c_{fenbufen}$ (mM)	$c_{DIMEB80}$ (mM)	$\Delta\delta_{free} - \Delta\delta_{complexed}$			
		H2,2’	H3,3’	H4,4’	H5
0.985	0.254	-0.074	-0.072	0.006	0.024
	0.508	-0.083	-0.082	0.008	0.029
	1.53	-0.179	-0.171	0.024	0.070
	2.54	-0.204	-0.195	0.023	0.095
	5.08	-0.213	-0.196	0.023	0.095
	10.2	-0.208	-0.195	0.024	0.096
	15.2	-0.202	-0.194	0.025	0.097
0.972	20.1	-0.195	-0.202	0.031	0.098

Table S8. Chemical shift change of fenbufen aromatic hydrogens induced by complexation with methyl-beta-cyclodextrin (DIMEB95).

$c_{fenbufen}$ (mM)	$c_{DIMEB95}$ (mM)	$\Delta\delta_{free} - \Delta\delta_{complexed}$			
		H2,2'	H3,3'	H4,4'	H5
1.038	0.220	-0.043	-0.043	0.001	0.012
	0.513	-0.097	-0.096	0.003	0.026
	1.32	-0.180	-0.175	0.015	0.060
	2.49	-0.213	-0.204	0.015	0.087
	4.98	-0.223	-0.205	0.015	0.087
	10.0	-0.221	-0.207	0.013	0.085
	15.1	-0.215	-0.206	0.014	0.086
	19.8	-0.210	-0.203	0.017	0.089

Table S9. Chemical shift change of fenbufen aromatic hydrogens induced by complexation with random methyl-beta-cyclodextrin (RAMEB).

$c_{fenbufen}$ (mM)	c_{RAMEB} (mM)	$\Delta\delta_{free} - \Delta\delta_{complexed}$			
		H2,2'	H3,3'	H4,4'	H5
1.038	0.220	-0.042	-0.044	-0.007	0.000
	0.513	-0.069	-0.072	0.002	0.015
	1.32	-0.125	-0.130	0.022	0.046
	2.49	-0.154	-0.159	0.032	0.063
	4.98	-0.168	-0.178	0.039	0.076
	10.0	-0.173	-0.179	0.037	0.084
	15.1	-0.174	-0.179	0.037	0.085
	19.8	-0.174	-0.180	0.037	0.094

Table S10. Chemical shift change of fenbufen aromatic hydrogens induced by complexation with hydroxypropyl-beta-cyclodextrin (HPBCyD(4.5)).

$c_{fenbufen}$ (mM)	$c_{HPBCyD(4.5)}$ (mM)	$\Delta\delta_{free} - \Delta\delta_{complexed}$				
		H1,1'	H2,2'	H3,3'	H4,4'	H5
1.038	0.226	-0.006	-0.032	-0.038	-0.009	-0.003
	0.564	0.002	-0.054	-0.067	-0.004	0.010
	1.02	0.012	-0.081	-0.099	0.002	0.026
	1.58	0.019	-0.101	-0.125	0.008	0.039
	2.59	0.026	-0.117	-0.145	0.013	0.050
	5.19	0.031	-0.127	-0.159	0.016	0.058
	10.3	0.032	-0.131	-0.163	0.017	0.060
	15.5	0.033	-0.132	-0.165	0.017	0.061
	20.3	0.032	-0.132	-0.165	0.016	0.061

Table S11. Chemical shift change of fenbufen aromatic hydrogens induced by complexation with hydroxypropyl-beta-cyclodextrin (HPBCyD(6.3)).

c_{fenbufen} (mM)	$c_{\text{HPBCyD(6.3)}}$ (mM)	$\Delta\delta_{\text{free}} - \Delta\delta_{\text{complexed}}$				
		H1,1'	H2,2'	H3,3'	H4,4'	H5
1.038	0.218	-0.006	-0.032	-0.039	-0.010	-0.003
	0.544	0.005	-0.056	-0.069	-0.004	0.012
	0.98	0.017	-0.082	-0.102	0.003	0.029
	1.52	0.027	-0.101	-0.127	0.010	0.044
	2.50	0.034	-0.116	-0.146	0.014	0.054
	5.00	0.039	-0.126	-0.159	0.018	0.062
	9.90	0.041	-0.129	-0.163	0.018	0.064
	14.9	0.040	-0.131	-0.166	0.017	0.064
	19.6	0.040	-0.132	-0.166	0.017	0.064

Table S12. The measured ellipticity of fenoprofen by the addition of different amount of beta-cyclodextrin (BCyD) and randomly methylated methyl-beta-cyclodextrin (CRYSMEB).

c_{fenoprof} (mM)	c_{BCyD} (mM)	$\theta_{274.6\text{ nm}}$ (mdeg)	$\theta_{280.8\text{ nm}}$ (mdeg)	$c_{\text{fenoprof.}}$ (mM)	c_{CRYSMEB} (mM)	$\theta_{274.6\text{ nm}}$ (mdeg)	$\theta_{280.8\text{ nm}}$ (mdeg)
0.421	0	0	0	0.406	0	0	0
	0.199	1.52	1.97		0.240	1.05	1.30
	0.399	2.18	2.76		0.480	1.85	2.13
	0.600	2.72	3.36		0.720	2.29	2.59
	1.20	5.61	6.61		1.44	3.66	3.92
	1.99	6.97	8.14		1.92	3.93	4.21
	4.00	8.70	10.12		3.60	4.77	4.94
	6.00	8.72	10.09		4.80	5.14	5.36
	8.00	8.80	10.22		9.60	5.64	5.81

Table S13. The measured ellipticity of fenoprofen by the addition of different amount of random methyl-beta-cyclodextrin (RAMEB) and methyl-beta-cyclodextrin (DIMEB50)

c_{fenoprof} (mM)	c_{RAMEB} (mM)	$\theta_{276.8\text{ nm}}$ (mdeg)	$\theta_{284.8\text{ nm}}$ (mdeg)	c_{fenoprof} (mM)	c_{DIMEB50} (mM)	$\theta_{262.9\text{ nm}}$ (mdeg)	$\theta_{276.9\text{ nm}}$ (mdeg)	$\theta_{284.7\text{ nm}}$ (mdeg)
0.398	0.000	0	0	0.406	0	0	0	0
	0.235	0.863	0.875		0.236	0.200	0.388	0.443
	0.471	1.08	1.19		0.473	0.270	0.803	0.733
	0.706	1.32	1.33		0.709	0.552	1.14	1.16
	1.41	1.68	1.78		1.42	0.927	1.67	1.66
	1.88	1.83	1.92		1.89	1.16	1.86	1.80
	3.53	2.31	2.48		3.55	1.37	2.05	2.02
	4.71	2.30	2.46		4.73	1.73	2.16	2.15
	9.42	2.46	2.56		9.46	2.18	2.12	2.20

Table S14. The measured ellipticity of fenoprofen by the addition of different amount of methyl-beta-cyclodextrins (DIMEB80, DIMEB95)

$c_{fenoprof}$ (mM)	$c_{DIMEB80}$ (mM)	$\theta_{262.9\ nm}$ (mdeg)	$\theta_{276.9\ nm}$ (mdeg)	$\theta_{284.0\ nm}$ (mdeg)	$c_{fenoprof}$ (mM)	$c_{DIMEB90}$ (mM)	$\theta_{263.0\ nm}$ (mdeg)	$\theta_{275.5\ nm}$ (mdeg)	$\theta_{284.0\ nm}$ (mdeg)
0.409	0.000	0	0	0	0.409	0	0	0	0
	0.237	0.143	0.541	0.631		0.240	0.148	0.522	0.656
	0.475	0.451	0.904	0.996		0.480	0.528	0.834	0.968
	0.712	0.550	1.09	1.12		0.720	0.708	1.12	1.20
	1.42	1.091	1.71	1.71		1.44	0.981	1.52	1.55
	1.90	1.13	1.73	1.72		1.92	1.20	1.71	1.69
	3.56	1.47	1.89	1.80		3.60	1.33	1.67	1.64
	4.75	1.56	1.78	1.75		4.80	1.57	1.71	1.64
	9.49	1.77	1.37	1.28		9.60	1.66	1.19	1.13

Table S15. The measured ellipticity of fenoprofen by the addition of different amount of hydroxypropyl-beta-cyclodextrins (HPBCyD(4.5), HPBCyD(6.3)).

$c_{fenoprof}$ (mM)	$c_{HPBCyD(4.5)}$ (mM)	$\theta_{265.1\ nm}$ (mdeg)	$\theta_{271.1\ nm}$ (mdeg)	$c_{fenoprof}$ (mM)	$c_{HPBCyD(6.3)}$ (mM)	$\theta_{271.8\ nm}$ (mdeg)	$\theta_{278.2\ nm}$ (mdeg)
0.421	0.000	0	0	0.406	0.000	0	0
	0.242	0.638	0.563		0.238	0.629	0.564
	0.484	0.77	0.81		0.475	1.08	1.01
	0.727	1.20	1.17		0.713	1.08	0.96
	1.45	1.64	1.63		1.43	1.79	1.51
	1.94	1.84	1.82		1.90	2.09	1.75
	3.63	2.34	2.19		3.57	2.48	2.03
	4.84	2.30	2.18		4.75	2.50	2.03
	9.69	2.42	2.31		9.51	2.71	2.19

Table S16. Chemical shift change of fenoprofen aromatic hydrogens induced by complexation with beta-cyclodextrin (BCyD)

$c_{fenoprofen}$ (mM)	c_{BCyD} (mM)	$\Delta\delta_{free} - \Delta\delta_{complexed}$					
		H6,6'	H3	H7	H2	H5,5'	H4
0.421	0.199	-0.001	-0.006	0.006	0.010	-0.025	-0.053
	0.399	-0.003	-0.011	0.010	0.017	-0.047	-0.098
	0.60	-0.002	-0.009	0.008	0.014	-0.036	-0.077
	1.20	-0.004	-0.021	0.023	0.036	-0.095	-0.204
	1.99	-0.006	-0.027	0.028	0.046	-0.119	-0.257
	3.99	-0.007	-0.032	0.033	0.053	-0.141	-0.304
	5.98	-0.007	-0.034	0.036	0.058	-0.151	-0.328
	7.98	-0.007	-0.035	0.038	0.059	-0.155	-0.336

Table S17. Chemical shift change of fenoprofen aromatic hydrogens induced by complexation with randomly methylated methyl-beta-cyclodextrin (CRYSMEB)

$c_{\text{fenoprofen}}$ (mM)	c_{CRYSMEB} (mM)	$\Delta\delta_{\text{free}} - \Delta\delta_{\text{complexed}}$					
		H3	H7	H2	H5,5'	H1	H4
0.406	0.240	-0.013	0.010	0.006	-0.021	-0.002	-0.065
	0.480	-0.024	0.016	0.010	-0.040	-0.005	-0.116
	0.72	-0.034	0.021	0.013	-0.055	-0.008	-0.160
	1.44	-0.051	0.030	0.020	-0.082	-0.010	-0.238
	1.92	-0.059	0.035	0.023	-0.093	-0.012	-0.273
	3.60	-0.069	0.086	0.026	-0.106	-0.015	-0.328
	4.80	-0.076	0.043	0.027	-0.118	-0.015	-0.344
	9.60	-0.081	0.047	0.030	-0.128	-0.016	-0.376

Table S18. Chemical shift change of fenoprofen aromatic hydrogens induced by complexation with methyl-beta-cyclodextrin (DIMEB50)

$c_{\text{fenoprofen}}$ (mM)	c_{DIMEB50} (mM)	$\Delta\delta_{\text{fre}} - \Delta\delta_{\text{complexed}}$						
		H6,6'	H3	H7	H2	H5,5'	H1	H4
0.406	0.236	0.007	-0.023	0.018	0.016	-0.023	-0.014	-0.099
	0.473	0.009	-0.033	0.025	0.022	-0.033	-0.020	-0.143
	0.71	0.015	-0.057	0.043	0.038	-0.056	-0.034	-0.241
	1.42	0.021	-0.084	0.061	0.055	-0.079	-0.049	-0.349
	1.89	0.024	-0.088	0.066	0.061	-0.089	-0.055	-0.386
	3.55	0.027	-0.105	0.079	0.072	-0.103	-0.064	-0.453
	4.73	0.028	-0.110	0.082	0.075	-0.107	-0.067	-0.476
	9.46	0.030	-0.114	0.086	0.080	-0.112	-0.069	-0.505

Table S19. Chemical shift change of fenoprofen aromatic hydrogens induced by complexation with methyl-beta-cyclodextrin (DIMEB80)

$c_{\text{fenoprofen}}$ (mM)	c_{DIMEB80} (mM)	$\Delta\delta_{\text{free}} - \Delta\delta_{\text{complexed}}$						
		H6,6'	H3	H7	H2	H5,5'	H1	H4
0.409	0.237	0.007	-0.032	0.025	0.018	-0.041	-0.023	-0.140
	0.475	0.009	-0.048	0.036	0.027	-0.060	-0.034	-0.208
	0.71	0.012	-0.060	0.045	0.034	-0.076	-0.043	-0.263
	1.42	0.016	-0.084	0.063	0.049	-0.103	-0.059	-0.369
	1.90	0.014	-0.092	0.067	0.047	-0.111	-0.063	-0.402
	3.56	0.018	-0.105	0.075	0.063	-0.124	-0.070	-0.466
	4.75	0.019	-0.103	0.077	0.063	-0.131	-0.070	-0.469
	9.49	0.017	-0.103	0.077	0.067	-0.132	-0.073	-0.489

Table S20. Chemical shift change of fenoprofen aromatic hydrogens induced by complexation with methyl-beta-cyclodextrin (DIMEB95)

$c_{fenoprofen}$ (mM)	$c_{DIMEB95}$ (mM)	$\Delta\delta_{free} - \Delta\delta_{complexed}$						
		H6,6’	H3	H7	H2	H5,5’	H1	H4
0.409	0.240	0.005	-0.027	0.021	0.015	-0.036	-0.021	-0.120
	0.480	0.008	-0.046	0.033	0.026	-0.056	-0.032	-0.200
	0.720	0.012	-0.061	0.046	0.035	-0.077	-0.042	-0.270
	1.44	0.015	-0.084	0.062	0.049	-0.102	-0.059	-0.369
	1.92	0.016	-0.092	0.067	0.048	-0.110	-0.062	-0.403
	3.60	0.018	-0.096	0.076	0.062	-0.125	-0.070	-0.458
	4.80	0.017	-0.111	0.076	0.068	-0.125	-0.075	-0.496
	9.60	0.018	-0.113	0.076	0.068	-0.132	-0.072	-0.491

Table S21. Chemical shift change of fenoprofen aromatic hydrogens induced by complexation with random methyl-beta-cyclodextrin (RAMEB)

$c_{fenoprofen}$ (mM)	c_{RAMEB} (mM)	$\Delta\delta_{free} - \Delta\delta_{complexed}$						
		H6,6’	H3	H7	H2	H5,5’	H1	H4
0.406	0.238	0.009	-0.012	0.012	0.013	-0.020	0.002	-0.070
	0.475	0.013	-0.021	0.022	0.022	-0.037	0.003	-0.126
	0.713	0.016	-0.028	0.029	0.030	-0.050	0.009	-0.168
	1.43	0.022	-0.040	0.042	0.043	-0.070	0.003	-0.244
	1.90	0.025	-0.045	0.048	0.049	-0.080	0.012	-0.274
	3.57	0.028	-0.054	0.056	0.057	-0.097	0.012	-0.324
	4.75	0.028	-0.057	0.058	0.060	-0.101	0.013	-0.339
	9.51	0.030	-0.060	0.062	0.064	-0.108	0.014	-0.363

Table S22. Chemical shift change of fenoprofen aromatic hydrogens induced by complexation with hydroxypropyl-beta-cyclodextrin (HPBCyD(4.5))

$c_{fenoprofen}$ (mM)	$c_{HPBCyD(4.5)}$ (mM)	$\Delta\delta_{free} - \Delta\delta_{complexed}$					
		H6,6’	H3	H7	H2	H5,5’	H4
0.421	0.242	0.003	-0.013	0.009	0.009	-0.021	-0.066
	0.484	0.007	-0.022	0.018	0.017	-0.037	-0.119
	0.727	0.010	-0.030	0.024	0.024	-0.050	-0.160
	1.45	0.015	-0.044	0.036	0.035	-0.069	-0.236
	1.94	0.017	-0.050	0.041	0.039	-0.081	-0.268
	3.63	0.021	-0.058	0.050	0.047	-0.095	-0.318
	4.84	0.022	-0.061	0.052	0.050	-0.100	-0.335
	9.69	0.024	-0.066	0.057	0.054	-0.109	-0.361

Table S23. Chemical shift change of fenoprofen aromatic hydrogens induced by complexation with hydroxypropyl-beta-cyclodextrin (HPBCyD(6.3))

$c_{fenoprofen}$ (mM)	$c_{HPBCyD(6.3)}$ (mM)	$\Delta\delta_{free} - \Delta\delta_{complexed}$					
		H6,6’	H3	H7	H2	H5,5’	H4
0.398	0.235	0.016	-0.025	0.021	0.012	-0.013	-0.095
	0.471	0.024	-0.045	0.035	0.020	-0.023	-0.167
	0.706	0.031	-0.058	0.046	0.027	-0.031	-0.216
	1.41	0.043	-0.081	0.066	0.039	-0.044	-0.314
	1.88	0.049	-0.093	0.076	0.044	-0.050	-0.355
	3.53	0.057	-0.113	0.089	0.052	-0.058	-0.420
	4.71	0.060	-0.117	0.094	0.055	-0.062	-0.439
	9.42	0.063	-0.125	0.101	0.059	-0.067	-0.470
